# Molecular detection of *Coxiella burnetii* from the formalin-fixed tissues of Q fever patients with acute hepatitis

**DOI:** 10.1371/journal.pone.0180237

**Published:** 2017-07-03

**Authors:** Young-Rock Jang, Yong Shin, Choong Eun Jin, Bonhan Koo, Se Yoon Park, Min-Chul Kim, Taeeun Kim, Yong Pil Chong, Sang-Oh Lee, Sang-Ho Choi, Yang Soo Kim, Jun Hee Woo, Sung-Han Kim, Eunsil Yu

**Affiliations:** 1 Departments of Infectious Diseases, Asan Medical Center, University of Ulsan College of Medicine, Seoul, Republic of Korea; 2 Division of Infectious Disease, Department of Internal Medicine, Gil Medical Center, Gachon University College of Medicine, Incheon, Republic of Korea; 3 Department of Convergence Medicine, Asan Medical Center, University of Ulsan College of Medicine, Seoul, Republic of Korea; 4 Division of Infectious Diseases, Department of Internal Medicine, Soonchunhyang University Seoul Hospital, Soonchunhyang University College of Medicine, Seoul, Republic of Korea; 5 Department of Pathology, Asan Medical Center, University of Ulsan College of Medicine, Seoul, Republic of Korea; University of Texas Medical Branch, UNITED STATES

## Abstract

**Background:**

Serologic diagnosis is one of the most widely used diagnostic methods for Q fever, but the window period in antibody response of 2 to 3 weeks after symptom onset results in significant diagnostic delay. We investigated the diagnostic utility of Q fever PCR from formalin-fixed liver tissues in Q fever patients with acute hepatitis.

**Methods:**

We reviewed the clinical and laboratory data in patients with Q fever hepatitis who underwent liver biopsy during a 17-year period, and whose biopsied tissues were available. We also selected patients who revealed granuloma in liver biopsy and with no Q fever diagnosis within the last 3 years as control. Acute Q fever hepatitis was diagnosed if two or more of the following clinical, serologic, or histopathologic criteria were met: (1) an infectious hepatitis-like clinical feature such as fever (≥ 38°C) with elevated hepatic transaminase levels; (2) exhibition of a phase II immunoglobulin G (IgG) antibodies titer by IFA of ≥ 1:128 in single determination, or a four-fold or greater rise between two separate samples obtained two or more weeks apart; (3) histologic finding of biopsy tissue showing characteristic fibrin ring granuloma.

**Results:**

A total of 11 patients with acute Q fever hepatitis were selected and analyzed. Of the 11 patients, 3 (27%) had exposure to zoonotic risk factors and 7 (63%) met the serologic criteria. Granulomas with either circumferential or radiating fibrin deposition were observed in 10 cases on liver biopsy and in 1 case on bone marrow biopsy. 8 (73%) revealed positive *Coxiella burnetii* PCR from their formalin-fixed liver tissues. In contrast, none of 10 patients with alternative diagnosis who had hepatic granuloma revealed positive *C*. *burnetii* PCR from their formalin-fixed liver tissues.

**Conclusions:**

Q fever PCR from formalin-fixed liver tissues appears to be a useful adjunct for diagnosing Q fever hepatitis.

## Introduction

Q fever occurs in humans when small-particle aerosols containing *Coxiella burnetii* are inhaled. The most commonly identified sources of Q fever are farm animals such as cattle, goats, and sheep. Recently, household cats and dogs have also been reported as potential sources of urban outbreaks [1]. Clinical manifestations of Q fever are varied and non-specific: in acute cases, Q fever often presents as pneumonia or hepatitis, and in chronic cases, endocarditis is most often observed.

Serological test remains the method of choice for diagnosing *C*. *burnetii* infection as it is easy to establish and widely applicable. However, antibodies are detected only after 2–3 weeks from the onset of disease making a diagnosis of Q fever too slow in most clinical settings [1]. Another method of direct detection of C. *burnetii* is cell culture, but this method requires biosafety level 3 (BSL-3) laboratories and has a varying degree of sensitivity [2]. Recently, polymerase chain reaction (PCR) techniques have been developed for Q fever testing and successfully employed to detect DNA in both cell cultures and clinical samples [3]. However, there are limited data on Q fever PCR from formalin-fixed tissues, especially liver biopsy tissues. We thus investigated the diagnostic utility of Q fever PCR from formalin-fixed liver tissues in Q fever patients with acute hepatitis.

## Materials and methods

### Study patients

We retrospectively reviewed the clinical and laboratory data of diagnosed acute Q fever hepatitis patients who underwent liver biopsy in our institution from May 2000 to December 2016, and whose biopsied tissues were available. Acute Q fever hepatitis was diagnosed if 2 of the following clinical, serologic, or histopathologic criteria were met: (1) An infectious hepatitis-like clinical feature such as fever (≥ 38°C) with elevated hepatic transaminase levels, (2) Exhibition of a phase II immunoglobulin G (IgG) antibodies titer by IFA of ≥ 1:128 in single determination or a four-fold or greater rise between two separate samples obtained two or more weeks apart, (3) histologic finding of biopsy tissue showing characteristic fibrin ring granuloma. As controls, we tested biopsy liver tissues from the patients with confirmed alternative diagnoses who underwent liver biopsy between 2013 and 2016 and in whom their histologic findings of biopsy tissues showed granuloma. Controls were utilized to determine the specificity of PCR assay and to detect any cross-reaction with samples from patients with unrelated disease. Verbal and written consent were obtained from the study participants. This study was approved by the Institutional Review Boards of Asan Medical Center (2016–0748).

### Molecular methods

#### (1) DNA extraction

To detect *C*. *burnetii*, DNA was extracted from the formalin-fixed liver tissues of Q fever patients with acute hepatitis. For each paraffin block, five slices of sections (5μm thick) were cut and placed in microtube. First, xylene was added to each tube and centrifuged (12,000rpm, 5min), and the supernatant was discarded. This procedure was carried out for three times. The specimens were then rehydrated through graded ethanol and centrifuged in each washing step. Finally, the tubes were kept open for the remaining ethanol evaporation. DNA extraction was performed using QIAamp DNA Mini Kit (Qiagen, Hilden, Germany) according to the manufacturer’s instruction with minor modifications. For tissue digestion, AL buffer and proteinase K were added, and samples remained in a water bath for 18h. Washing steps with these buffers were done twice, and samples were eluted in 100 μl of TAE buffer and stored at −20°C until use.

#### (2) PCR assay

End-point PCR was performed to detect the *C*. *burnetii* from the tissues. The gene target was determined to be that derived from the transposase gene insertion element IS1111a of *C*. *burnetii* isolate LBCE 13265 (NCBI Nr. KT 965031.1). The forward (5’-GAGCGAACCATTGGTATCG-3’) and reverse (5’-TTTAACAGCGCTTGAACGT-3’) primers were synthesized at the usual length of around 24 bp. The end-point PCR process consisted of an initial denaturation step at 95°C for 15 min; 45 cycles of 95°C for 30s, 57°C for 30s, and 72°C for 30s; and a final elongation step at 72°C for 7 min. 5 μL of DNA were amplified in a total volume of 25 μL containing 10X PCR buffer (Qiagen), 2.5 mM MgCl_2_, 0.25 mM deoxynucleotide triphosphate, 25 pmol of each primer, and 1 unit of Taq DNA polymerase (Qiagen). Gel electrophoresis was used to separate PCR products on a 2% agarose gel containing ethidium bromide (EtBr), and visualized using a GelDoc System (Clinx Science Instruments).

#### (3) Sequencing of PCR product and sequence analysis

For direct sequencing of DNA, all DNA samples were amplified with primers for *C*. *burnetii*, and then purified by using Expin PCR SV (GeneAll, Korea). The purified samples were directly sequenced using BigDyeTerminal chemistry with the forward primer of Q-fever_IS111. We used Macrogen sequencing service (Macrogen Inc. Korea), through which the DNA sequencing reactions were electrophoresed on ABI's3730XL DNA Analyzers (Applied Biosystems, USA), which produces read lengths of 800–1000 bases.

## Results

A total of 11 patients with acute Q fever hepatitis were selected and analyzed. Baseline clinical characteristics and laboratory findings are shown in Table 1. All cases were males, with median patient age (range) of 50 years (29–70 years). All patients had acute onset fever, and none had history of chronic liver disease. Three patients had exposure to zoonotic risk factors such as cows and dogs. All patients revealed abnormal liver function tests. One patient (case 2) with severe jaundice showed marked elevation of total and direct bilirubin levels up to 23-fold higher than reference levels, whereas others revealed only slight increase in liver enzymes. No patients showed bacterial growth on blood cultures. Antibodies for leptospirosis, brucellosis, hepatitis A, B, and C, as well as autoantibodies including anti-nuclear antibody, anti-double strand DNA antibody, and anti-neutrophilic cytoplasmic antibody were all negative.

**Table 1:** Clinical and laboratory findings and PCR results in 11 case patients with acute Q fever hepatitis.

Case number	Age/ sex	Symptoms	Predisposing factor	AST / ALT / γ-GTP, IU/L	Total bilirubin, mg/dL	IFA titer		Case definition	Treatment	Pathologic findings of liver biopsy	IS1111 PCR result from liver tissues	IS1111 PCR result from blood samples
						Phase 2 IgG	Phase 2 IgM					
1	70/M	Diarrhea	Gastric cancer, History of tuberculous pleurisy	58/33/74/51	1.9	1:1024	1:152	Clinical / Serologic / Pathologic	Doxycycline	Typical fibrin ring granuloma	Positive*	Not available
2	62/M	Jaundice	None	49/12/569/57	23	Not done	Not done	Clinical / Pathologic	Doxycycline Rifampin Ciprofloxacin	Periportal fibrosis†	Positive*	Not available
3	33/M	Fever	None	80/199/160/115	1.6	1:152	1:256	Clinical / Serologic / Pathologic	Doxycycline	Typical fibrin ring granuloma	Negative	Not available
4	52/M	Fever	History of pulmonary tuberculosis	153/206/213/471	1.4	1:152	<1:16	Clinical / Serologic / Pathologic	Doxycycline	Typical fibrin ring granuloma	Negative	Not available
5	59/M	Fever	History of pulmonary tuberculosis	102/104/103/330	0.9	1:64	1:32	Clinical / Pathologic	Doxycycline	Typical fibrin ring granuloma	Positive*	Not available
6	58/M	Fever	Diabetes mellitus	139/88/273/229	16.6	1:152	1:64	Clinical / Serologic / Pathologic	Doxycycline	Typical fibrin ring granuloma	Negative	Not available
7	29/M	Fever	None	130/140/70/95	1.1	>1:2048	> 1:2048	Clinical / Serologic / Pathologic	Doxycycline	Typical fibrin ring granuloma	Positive*	Not available
8	47/M	Fever	Hyperlipidemia	80/92/65/58	0.7	1:128	< 1:16	Clinical / Serologic / Pathologic	Doxycycline	Non-caseating granuloma, portal inflammation	Positive*	Not available
9	54/M	Fever	Fatty liver	128/75/105/113	4.6	1:1024	1:1024	Clinical / Serologic / Pathologic	Doxycycline	Typical fibrin ring granuloma	Positive*	Positive*
10	45/M	Fever	None	118/250/153/185	0.4	< 1:16	1:32	Clinical / Pathologic	Doxycycline	Non-caseating granuloma, portal inflammation	Positive*	Positive*
11	37/M	Fever	None	43/65/96/82	2.1	> 1:2048	1:2048	Clinical / Serologic / Pathologic	Doxycycline	Typical fibrin ring granuloma	Positive*	Negative

* The sequencing data from all positive samples revealed > 99% similarity of *Coxiella burnettii* isolated LBCE 13265 insertion sequencing IS1111A transposage gene, partial cds (KT965031.1).

† Characteristic fibrin ring granulomas were identified in bone marrow biopsy.

**Fig 1:**
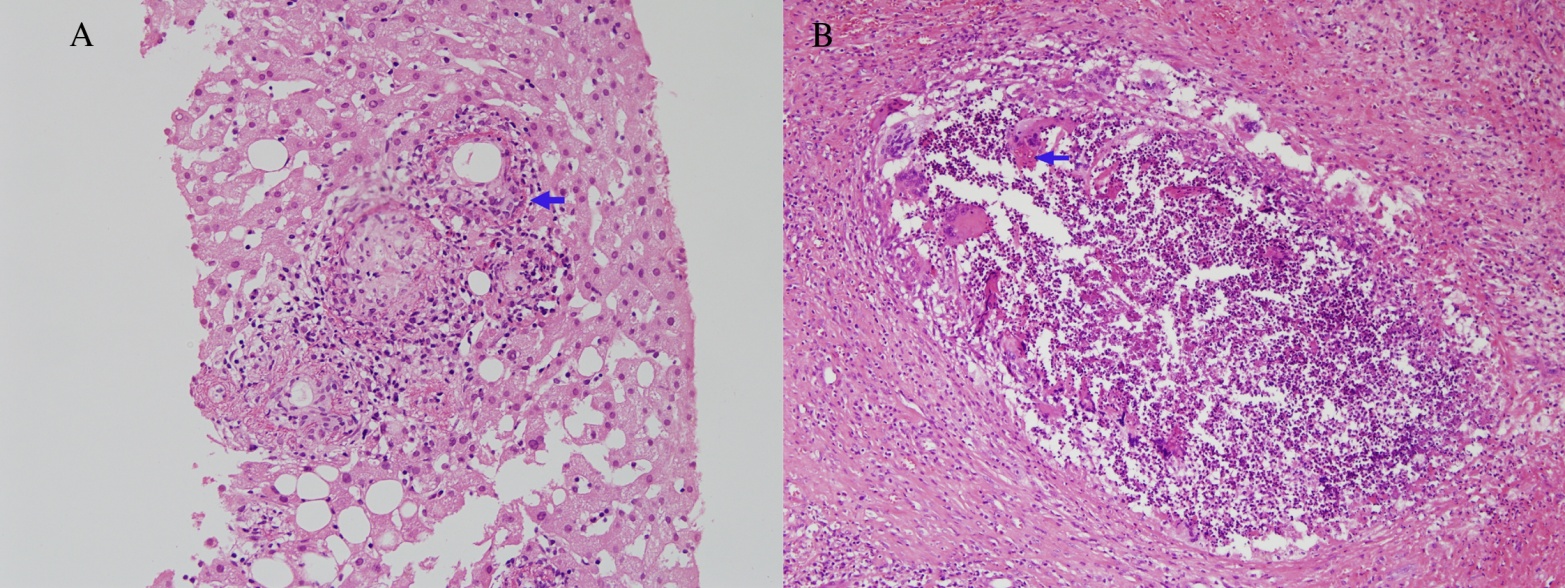
Representative photomicrographs of Q fever hepatitis (case no. 3 in case group, x200) and hepatic mucormycosis (case no. 6 in control group, x100). (A) Characteristic fibrin ring granulomas consisting of a central fat globule or epitheloid cells with fibrin ring (arrow) (B) A suppurative granuloma consists of multinucleated giant cells with fungal hyphae (arrow) and polymorphous lymphoid cell including eosinophils.

**Fig 2:**
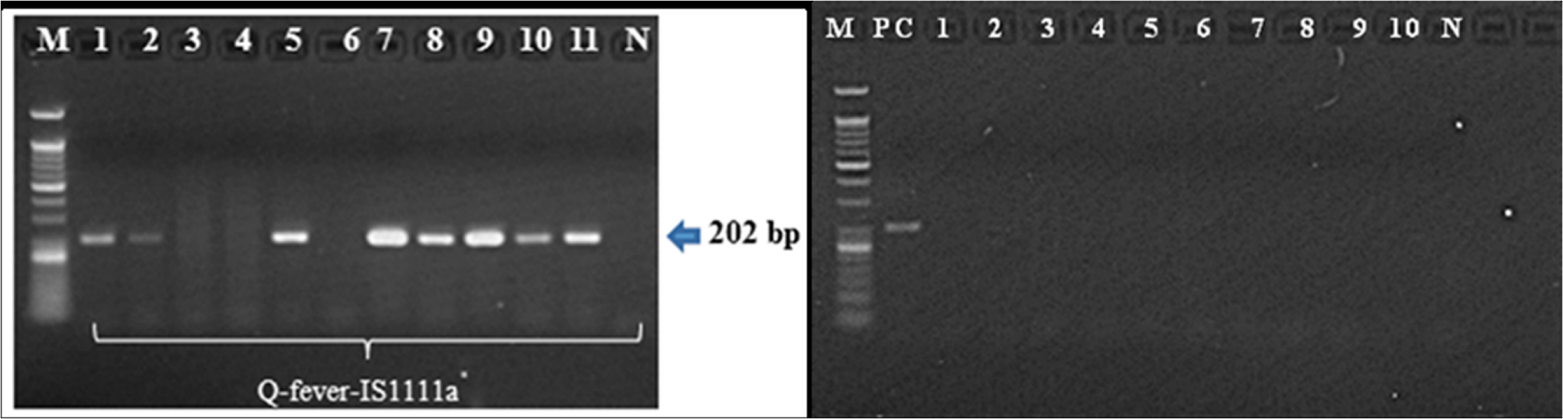
Agarose gel electrophoresis of *Coxiella burnetii IS1111a gene*. DNA amplification with Q-fever-IS1111a primers for the detection of *Coxiella burnetii*. Gel electrophoresis of 202 bp products by using end-point PCR. M: 50bp DNA size marker; 1–11: DNAs from the case patients with Q fever hepatitis and N: negative control (left). M: 50bp DNA size marker; PC: *C*. *burnetii* DNA control (case number 1 of case patient group); 1–10: DNAs from the control patients and N: negative control (right).

Granulomas with either circumferential or radiating fibrin deposition (Fig 1A) were observed in 10 cases on liver biopsy and 1 case on bone marrow biopsy (case 2). Of 11 patients, 8 (73%) revealed positive *C*. *burnetii* PCR results from their formalin-fixed liver biopsy specimen (Table 1). So, the sensitivity of *Coxiella burnetii* PCR was 73% (95% CI 43–90). All eight PCR products were sequenced; the corresponding data are summarized in Table 1, and an agarose gel profile of the PCR products is presented in Fig 2. Of the 3 patients (case 9, 10, 11) in whom the stored blood samples were available, 2 (case 9, 10) revealed positive *Coxiella burnetii* PCR from their blood samples. The time intervals from the onset of symptoms to blood sampling were 14, 10, and 15 days in case 9, 10, and 11, respectively. None of the aforementioned 3 patients received doxycycline before blood sampling.

As for control liver biopsy tissues, 10 patients with confirmed diagnosis unrelated to Q fever showing granuloma in liver biopsy tissue (Fig 1B) were evaluated to test if there were any cross-reaction. Three patients with hepatic tuberculosis, 2 patients with chronic disseminated candidiasis, 2 patients with primary biliary cirrhosis, 1 patient with hepatic mucormycosis, 1 patient with alcoholic hepatitis, and 1 patient with post-transplant lymphoproliferative disease. The clinical diagnosis and PCR results for these 10 control patients are summarized in Table 2. Of these 10 control patients, none revealed positive *C*. *burnetii* PCR from their live biopsy tissues. The agarose gel profile of PCR products are shown in Fig 2.

**Table 2:** Clinical and laboratory findings and PCR results in 10 control patients with hepatic granuloma.

Case number	Age/ sex	Symptoms	Predisposing factor	AST / ALT / γ-GTP, IU/L	Total bilirubin, mg/dL	Final diagnosis	Treatment	Pathologic findings of liver biopsy	IS1111 PCR result from liver tissues
1	46/M	Fever	None	184/135/417	4.5	Hepatic tuberculosis	Anti-tuberculous treatment	Chronic granulomatous inflammation	Negative
2	59/M	General weakness	None	17/19/ 109	0.6	Hepatic tuberculosis	Anti-tuberculous treatment	Chronic granulomatous inflammation with caseous necrosis	Negative
3	49/F	Fever	Acute myeloid leukemia	31/70/ 129	0.3	Chronic disseminated candidiasis	Anti-fungal treatment	Chronic granulomatous inflammation	Negative
4	60/M	Fever	Acute myeloid leukemia	15/13/79	1.4	Chronic disseminated candidiasis	Anti-fungal treatment	Necrotizing granulomas	Negative
5	56/M	Fever	DM	73/23/ 193	5.3	Alcoholic hepatitis	Corticosteroid	Non-necrotizing micro-granulomas	Negative
6	47/F	Fever	Acute myeloid leukemia	38/53/ 201	4.2	Hepatic mucormycosis	Anti-fungal treatment	Necrotizing granulomatous inflammation	Negative
7	55/M	Abdominal discomfort	None	78/54/ 468	0.2	Primary biliary cholangitis	Ursodesoxycholic acid	Portal inflammation with ill-defined microgranuloma.	Negative
8	15/F	General weakness	Liver transplantation recipient	218/260/91	0.6	Post-transplant lymphoproliferative disorder	Corticosteroid	Diffuse sinusoidal lymphocytosis with microgranuloma	Negative
9	56/F	Palpitation	None	22/9/130	0.5	Primary biliary cholangitis	Ursodesoxycholic acid	Noncaseating granuloma	Negative
10	33/M	Abdominal pain	None	129/41/ 40	0.5	Hepatic tuberculosis	Anti-tuberculous treatment	Chronic granulomatous inflammation with caseous necrosis	Negative

## Discussion

In this study, we found that PCR targeting for C. *burnetii* IS1111 multicopy sequence from formalin-fixed, paraffin-embedded liver tissues are useful for diagnosing patients with suspected Q fever. A previous study showed that PCR has been successfully applied for the direct detection of *C*. *burnetii* in 12 cardiovascular biopsy specimens [4]. It has been also reported that PCR for Q fever can be also performed on cerebrospinal fluid, pleural fluid, bone marrow, bone biopsies, liver biopsies, milk, placenta, and fetal tissue [5–7]. However, there are limited data on whether PCR on formalin-fixed liver tissue can be used for diagnosing Q fever hepatitis.

All patients in the present study had fever and feature of acute hepatitis, and underwent percutaneous or transjugular liver biopsy. Patients with fever of unknown origin (FUO) occasionally undergo liver biopsy to establish a final diagnosis [8, 9]; therefore, liver biopsy specimens are frequently obtained in FUO patients with hepatitis, and it is important this sample is available for direct diagnosis. Acute Q fever hepatitis is one of the most common manifestations of *C*. *burnetii* infection [10, 11]. In patients with FUO related to Q fever, liver biopsy reveals a typical fibrin ring type granuloma [12, 13], which has a central lipid vacuole surrounded by a dense fibrin ring. These granulomas are highly suggestive of Q fever but not definitive, because they are also seen in cases of Hodgkin’s disease, infectious mononucleosis, typhoid fever, and allopurinol hypersensitivity [12]. After acute infection of *C*. *burnetii*, the bacterial DNA remains for an extended period in several tissues, particularly in the bone marrow and liver; in these tissues, DNA can be detected up to decades after infection, even in the absence of serological evidence for *C*. *burnetii* infection [5, 14]. In this context, our data suggest that Q fever PCR from the liver tissue can be a useful adjunct for differential diagnosis in febrile patients with fibrin ring type granulomas. One patient showed a discrepancy of PCR results from blood sample (negative PCR result) and liver tissue (positive PCR result); such discrepancy of PCR results between blood samples and the tissues has also been reported in a previous study [4]. One possible explanation for this observation is that whereas blood coxiellemia persists in the acute phase (first 2 weeks from the symptom onset) but disappears 24–48 hours after antibiotic treatment [15, 16], organ tissues are able to harbor DNA and antigen up to decades after *C*. *burnetii* infection [5, 7, 14]. Further studies are needed on the comparison of diagnostic usefulness according to the clinical specimens.

This study has several limitations. First, we did not exam the *C*. *burnetii* DNA by PCR from fresh liver biopsy specimen. To diagnose acute Q fever hepatitis, the PCR from fresh liver biopsy specimen would be more useful than that from formalin-fixed liver tissues. However, our positive data on the *C*. *burnetii* DNA by PCR from the formalin-fixed liver tissues warrant the further prospective study on the usefulness of the *C*. *burnetii* DNA by PCR from fresh liver biopsy specimen in patients with suspicious acute Q fever hepatitis. Second, we included data on patients who had similar pathology with Q fever hepatitis used as controls. Because some control patients might have different clinical presentations from the patients with Q fever hepatitis, they are not representative of the population to which the test will be applied in clinical practice. However, this study provides the useful information in the setting of the pathologic findings suggesting Q fever hepatitis.

In conclusion, Q fever PCR from formalin-fixed liver tissues appears to be a useful adjunct for diagnosing Q fever hepatitis. This diagnostic method might prove helpful in determining Q fever hepatitis in patients with FUO who have fibrin-ring type granuloma, especially in the absence of serologic evidence in acute phase of Q fever and in cases in convalescent period, during which a direct detection for microbiologic evidence using PCR or culture from blood samples is impossible.
